# Effect of acupuncture on essential hypertension: A protocol for systematic review and meta-analysis

**DOI:** 10.1097/MD.0000000000025572

**Published:** 2021-04-16

**Authors:** Jing Yu, Yi Wei, Yang Jing, Yongli Gao

**Affiliations:** a West China School of Nursing; b Department of Central Medical Transportation, West China Hospital, Sichuan University; c Department of Cardiology, Sichuan Hospital of Integrated Traditional and Western Medicine; d Department of Emergency Medicine, West China Hospital, Sichuan University, Chengdu, China.

**Keywords:** acupuncture, hypertension, meta-analysis, protocol, systemic review

## Abstract

**Background:**

Essential hypertension is a risk factor for early cardiovascular disease and is a major preventable risk factor for premature death and disability worldwide. However, some antihypertensive drugs cannot be used for treatment because of their cost-effectiveness or side effects. Non-drug treatments for hypertension include weight loss, salt restriction, smoking cessation, alcohol withdrawal, and exercise, although these methods are difficult to maintain and to achieve. This study will investigate the efficacy and safety of acupuncture and moxibustion in the treatment of different grades of essential hypertension.

**Methods and analysis:**

A systematic search of the Cochrane, PubMed, EMBASE, CNKI, WanFang, VIP, and CBM databases will be performed, which will include randomized controlled trials on acupuncture for essential hypertension. The main results will include systolic and diastolic blood pressure before and after treatment, whereas the secondary outcomes will be efficacy rate and adverse events. The possible adverse reactions include dizziness, headache, edema, cough, nausea, electrolyte disorders, and hypotension. RevMan Manager 5.3 (Cochrane Collaboration) and STATA 16.0 software will be used to calculate mean deviation, standard deviation, confidence interval, and *P* values. For continuous variables, we will use the standardized mean difference of 95% confidence intervals as the summary statistics of the meta-analysis.

**Results:**

This study will assess the efficacy and safety of acupuncture for essential hypertension.

**Conclusions:**

Our study will determine the efficacy and safety of acupuncture in treating primary hypertension, and provide the basis for clinical decision-making.

**INPLASY registration number:**

INPLASY202130042.

## 1 Introduction

Essential hypertension is a risk factor for early cardiovascular disease and is a major preventable risk factor for premature death and disability worldwide.^[1,2]^ There are ∼1 billion patients with hypertension worldwide, with the number of adult patients expected to exceed 1.56 billion in 2025.^[3]^ Moreover, high blood pressure kills 940,000 people per year, and deaths from hypertension accounted for 17.4% of the total mortality rate in the United States in 2007.^[4,5]^ From 2011 to 2014, there were 85.7 million patients with hypertension in the United States, accounting for one-third of the entire adult population and two-third of the population >60 years old. Among them, the hypertension awareness rate was 84%, 75% of the patients received antihypertensive treatment, and only 50% of the patients’ blood pressure became effectively controlled.^[6]^ Between 2009 and 2010, 48% of patients with hypertension treated in the United States took more than one drug, and 40% of them still had poor blood pressure control.^[7]^ According to a survey, the number of adults with hypertension in China has reached 245 million, whereas the number of pre-hypertension cases has reached 435 million.^[8]^ Nearly half of all adults aged 35 to 75 years have high blood pressure, less than a third of patients are receiving antihypertensive treatment, and <1/12th have good blood pressure control.^[9]^ Blood pressure, known as “refractory hypertension,” remains uncontrolled in 0.5% to 1.4% of patients with hypertension, even with the use of five or more antihypertensive drugs; this has recently been identified as a new phenotype of failure in antihypertensive treatment, with a prevalence of between 3% and 31% of all patients.^[10]^ Patients with elevated blood pressure are at a higher risk of myocardial infarction, stroke, and congestive heart failure.^[11]^ For every 10 mm Hg, systolic blood pressure decreases the risk of heart failure by 28%, stroke by 27%, coronary heart disease by 17%, and all-cause mortality by 13%.^[12]^ However, some antihypertensive drugs cannot be used for treatment because of their cost-effectiveness and side effects.^[13]^ In contrast, the non-drug treatments for hypertension include weight loss, salt restriction, smoking cessation, alcohol withdrawal, and exercise, although these methods are difficult to maintain.^[14]^ Therefore, many patients with hypertension need more effective, safe, and individualized treatments.^[15]^

Acupuncture and moxibustion have a more than 3000-year history in China. It is an ancient treatment method that is characterized by needling into a specific part of the human body and the matching of different techniques to produce a stimulus response.^[16]^ In recent decades, acupuncture has become the most popular and recognized complementary and alternative therapy in Western countries.^[17]^ Acupuncture may target the renin-angiotensin-aldosterone system, reducing sympathetic excitability of the kidney and regulating vasoactive substances, hence producing antihypertensive effects.^[18–20]^ A meta-analysis showed that there was no significant difference between acupuncture and moxibustion in terms of their effect on hypertension, and other research results show that acupuncture can significantly reduce blood pressure in patients taking antihypertensive drugs.^[21,22]^ The lack of high-quality randomized controlled studies on acupuncture and moxibustion for essential hypertension may lead to different results in systematic evaluation. Nevertheless, the current literature has been updated to enable us to conduct a systematic meta-analysis of existing randomized controlled trials to evaluate the efficacy and safety of acupuncture in the treatment of different grades of essential hypertension.

## 2 Methods

The study will follow the Preferred Reporting Items for Systematic Review and Meta-Analysis (PRISMA) 2015 statement.^[23]^ Enrollment was completed in March 2021 on the International Platform of Registered Systematic Review and Meta-Analysis Protocols (INPLASY), with registration number: INPLASY202130042. The platform can be accessed at https://inplasy.com/inplasy-2021-3-0042. The study did not require ethical approval because it did not involve participants’ information or violation of their privacy.

### 2.1 Research criteria

The inclusion criteria will be as follows:

1. randomized controlled trials;
2. the patient is clearly diagnosed with essential hypertension with systolic blood pressure ≥140 mm Hg and diastolic blood pressure ≥90 mm Hg, or is currently being treated with antihypertensive drugs;
3. the experimental group should have received multiple types of routine acupuncture, electroacupuncture, Yaoluo acupuncture, ear acupuncture, and others, without limiting the number of acupoints and time of acupuncture, as well as the oral or non-oral antihypertensive drugs; and
4. the control group should have received either sham acupuncture or no treatment.

In contrast, the exclusion criteria will be as follows:

1. non-randomized controlled studies;
2. zoopery;
3. lack of blood pressure follow-up data;
4. the control group received alternative treatments of other types with uncertain efficacy;
5. incomplete data extraction;
6. duplicate studies; and
7. reviews of literature.

The main results will include systolic and diastolic blood pressure before and after treatment.

The secondary outcomes will be efficacy rate and adverse events, which refers to the proportion of the number of people with diastolic blood pressure ≥10 mm Hg, diastolic blood pressure ≤90 mm Hg, or systolic blood pressuredrop ≥30 mm Hg, to the total number of people. The possible adverse reactions include dizziness, headache, edema, cough, nausea, electrolyte disorders, and hypotension.

### 2.2 Search strategy

A systematic search of Cochrane, PubMed, EMBASE, CNKI, WanFang, VIP, and the CBM databases will be performed, reviewing publications from the beginning of the database to February 2021, with no language restrictions. The English retrieval terms will be: “Hypertension” or “Blood pressure” and “Acupuncture” or “Electroacupuncture” or “pharmacoacupuncture” or “Auricular acupuncture.” The Chinese retrieval terms will be: “Zhen jiu” and “Gao Xue Ya.” The subject word and free word retrieval will be combined during search. Grey literature will also be searched to avoid literature omission.

### 2.3 Data collection and analysis

#### 2.3.1 Literature screening

**Figure 1:**
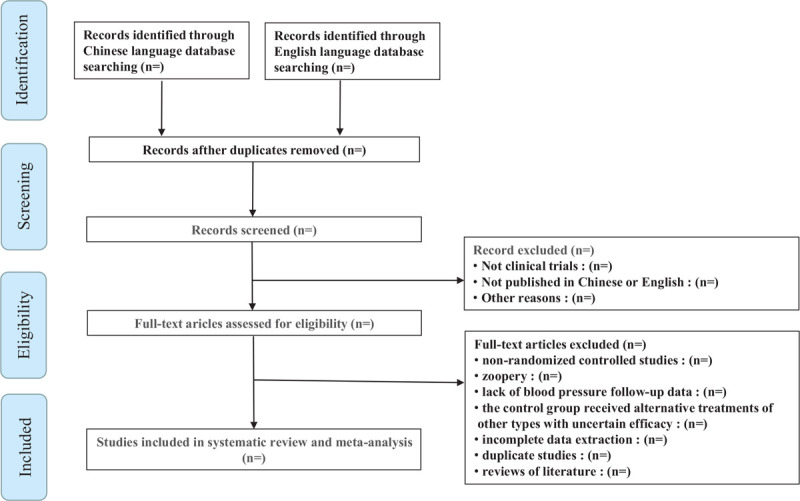
Flow chart of literature screening.

During the first screening, two researchers will look through the title of the literature, abstract, and other content, excluding those that obviously do not meet the criteria. Secondary screening will involve full-text reading of potentially qualified literature to further exclude unqualified literature. A third screening will be performed if the information in the literature cannot be clearly included, which will hence involve contacting the authors by mail, telephone, and other means to obtain relevant information. The details of the study selection and identification process will be presented in a flow chart (Fig. 1).

#### 2.3.2 Literature evaluation

The quality of the literature will be evaluated using the Cochrane Collaboration network bias risk assessment tool to determine if the following entries are included:

1. generation of random allocation schemes,
2. blindness for patients and doctors,
3. results evaluation of blind method,
4. is it hidden Tibetan distribution program,
5. is it a selective report,
6. the result is complete, and
7. is there any other bias.

The literature on therapy will be classified as A (full compliance with criteria, low likelihood of bias), B (partial compliance with criteria, moderate likelihood of bias), or C (total dissatisfaction, high likelihood of bias). Two researchers will independently perform the search and screening, and will evaluate the quality of the included literature. A third researcher could be involved to negotiate the decision in cases of disagreement.

#### 2.3.3 Data fetch

A uniform extraction form containing the following information will be developed: title, author, time of publication, design of the research scheme, sample size, missing persons, treatment method and course of treatment, curative effect index, outcome index, and adverse events. The data will be independently extracted by two researchers. A third researcher could be involved to negotiate the decision in cases of disagreement.

#### 2.3.4 Data deletion processing

The researchers will contact the authors by email for missing information not explicitly described in the studies to be included. If the continuous result data are not represented as means and standard deviations, we will try to recalculate the means and standard deviations as the first choice. Moreover, we will conduct a sensitivity analysis to address the potential impact of missing data. The potential impact of missing information on the final review results will be addressed in the discussion.

#### 2.3.5 Data analysis

RevMan Manager 5.3 (Cochrane Collaboration) and STATA 16.0 software will be used to calculate the mean deviation, standard deviation, confidence interval, and *P* value. For continuous variables, we will use the standardized mean difference of 95% confidence intervals as the summary statistics of the meta-analysis.

#### 2.3.6 Heterogeneity analysis

Heterogeneity among the included studies will be assessed using the chi-square and *I*^2^ tests. If *I*^2^ > 50%, the study will be considered highly heterogeneous, and a random-effects model will be used. In contrast, a fixed model will be used to analyze the data if *I*^2^ < 50%, which will indicate that there is no significant heterogeneity between the tests. When heterogeneity occurs, sensitivity analysis or meta-regression will be performed to assess the source of heterogeneity.

#### 2.3.7 Subgroup analysis

If there are enough randomized controlled trials, when there is significant heterogeneity in the trials. We will analyze the sub-groups according to age, country, sex, oral antihypertensive drugs, course of intervention, and type of intervention in the experimental and control groups. Intervention methods can be divided into routine acupuncture, electroacupuncture, drug collateral needle, and ear needle.

#### 2.3.8 Publication bias analysis

If enough randomized controlled trials are included (>10), funnel plots or Egger's and Begg's tests will be used to assess potential publication bias. If there is no deviation in the included studies, the points on the funnel plot will be symmetrically dispersed around the estimated true values of each independent study effect point, showing an inverted symmetrical funnel shape. If there is asymmetric funnel plot, the more obvious the asymmetry, the greater the degree of bias, which may overestimate the therapeutic effect.

#### 2.3.9 Sensibility analysis

Sensitivity analyses will be conducted in tests with sufficient data to test the robustness and reliability of the results where necessary. When there is significant heterogeneity, we will conduct a sensitivity analysis, and some low-quality or unblinded studies will be excluded.

#### 2.3.10 Ethics and communication

Because the study is a secondary analysis, it will not involve patient and public information, and hence does not require ethical review. The results of this study will be disseminated through peer-reviewed publications, journals, and academic exchanges.

## 3 Discussion

Many studies have been conducted on acupuncture and moxibustion in the treatment of hypertension, although the efficacy and safety of acupuncture and moxibustion on different types, grades, and risk stratifications of essential hypertension have not been clearly demonstrated. A systematic review and meta-analysis can be used to evaluate the efficacy and safety of exercise acupuncture in the treatment of essential hypertension and to provide a reference for the treatment of patients with this condition, according to PRISMA guidelines and reports. Nevertheless, this program could also have some limitations, such as the lack of high-quality randomized controlled studies. Due to the particularity of acupuncture treatment, it is difficult to achieve the same point, acupuncture manipulation, operation amplitude frequency, and operation time. Furthermore, the hidden distribution implementation description could also be unclear. Nevertheless, although the mechanism of acupuncture and moxibustion for hypertension is still unclear, many studies have shown that acupuncture and moxibustion may be related to the renin-angiotensin-aldosterone system, vascular endothelium, oxidative stress, neuroendocrine system, and other factors.^[24]^ In future research, we should conduct multi-center, large-sample studies, along with further optimization of acupoint compatibility and further improvement in the accuracy of acupuncture stimulation, to establish a more objective and systematic treatment, and to provide a reliable basis for further proving the efficacy of acupuncture and moxibustion in the treatment of essential hypertension.

## Author contributions

Conceptualization: Jing Yu; Methodology: Jing Yu; Project administration: Yongli Gao; Resources: Yongli Gao; Validation: Yang Jing; Writing – original draft: Jing Yu; Writing – review & editing: Yi Wei.

**Conceptualization:** Yu Jing, Yi Wei, Yongli Gao.

**Methodology:** Yu Jing, Yi Wei, Yongli Gao.

**Software:** Yu Jing.

**Writing – original draft:** Yu Jing, Yi Wei, Yang Jing, Yongli Gao.

**Writing – review & editing:** Yongli Gao.
